# *Schistosoma japonicum* infection downregulates house dust mite-induced allergic airway inflammation in mice

**DOI:** 10.1371/journal.pone.0179565

**Published:** 2017-06-14

**Authors:** Sugan Qiu, Xiaolin Fan, Yingying Yang, Panpan Dong, Wei Zhou, Yongliang Xu, Yonghua Zhou, Fukun Guo, Yi Zheng, Jun-Qi Yang

**Affiliations:** 1 Key Laboratory of National Health and Family Planning Commission on Parasitic Disease Control and Prevention, Jiangsu Provincial Key Laboratory on Parasite and Vector Control, Jiangsu Institute of Parasitic Diseases and Public Health Research Center of Jiangnan University, Wuxi, Jiangsu, China; 2 Division of Experimental Hematology and Cancer Biology, Cincinnati Children’s Hospital Research Foundation, Cincinnati, OH, United States of America; University of Cincinnati College of Medicine, UNITED STATES

## Abstract

The “hygiene hypothesis” is a theory try to explain the dramatic increases in the prevalence of autoimmune and allergic diseases over the past two to three decades in developed countries. According to this theory, reduced exposure to parasites and microorganisms in childhood is the main cause for the increased incidences of both T helper 1 (Th1)-mediated autoimmunity and Th2-mediated allergy. In this study, we investigated the impact of *Schistosoma japonicum* infection on the allergic airway inflammation induced by repeated intracheal inoculations of house dust mites (HDM), which is a Th17 and neutrophils dominant murine asthma model, mimicking severe asthma. We found that *S*. *japonicum* infection downregulated airway hyperresponsiveness. The infiltrating cells, Th17 and Th2 effector cytokines in the bronchoalveolar lavage (BAL) fluids and lungs were significantly reduced in the infected mice. Our findings indicated that *S*. *japonicum* infection was able to effectively inhibit host’s allergic airway inflammation, which may be related to the upregulated Treg cells upon infection. To our knowledge, it is the first study to reveal the impact of *S*. *japonicum* infection on house dust mite induced severe asthma. More in depth investigation is need to elucidate the underlying mechanisms.

## Introduction

In 1989, Strachan proposed the “hygiene hypothesis” to explain the dramatic increase in the prevalence of autoimmune and allergic diseases over the past two to three decades [1]. According to this hypothesis, a lack of early life exposure to microorganisms and parasites may cause an increased incidences of both T helper 1 (Th1)-mediated autoimmunity and Th2-mediated allergy [2–4]. Parasitic infections are becoming a major theme in the hygiene hypothesis [5]. Helminths, as long-lived parasites, possess their ability to manipulate host immunity to protect themselves from elimination and minimize severe pathology to the host [6]. Immunomodulation by parasitic helminths is a general and very conserved phenomenon [7].

Schistosomiasis is an immunopathogenic disorder. It is caused by blood flukes, of which there are three main species: *Schistosoma japonicum*, *S*. *mansoni*, and *S*. *haematobium*. The delayed-type hypersensitivity reactions against the schistosome eggs s trapped in the venules lead to the formation of circumoval granuloma in livers and intestines and subsequent fibrosis [8, 9]. Schistosomiasis is roughly divided into acute, chronic, and late phases upon infection progression. Th1 responses are elicited at early phase by the larval worms, succeeded by Th2 responses induced by the parasite eggs. Egg deposit in the tissues is a determining factor to drive Th2 response in *S*. *mansoni* and *S*. *japonicum* infections in mice [9, 10]. After infection progresses into chronic phase, usually 8–10 weeks (wks) post infection in mice, immunomodulation phenomenon occurs. The latter not only downregulates granuloma formation and other parasite-related immune responses, but also significantly affects host’s systemic immune responses. The underlying mechanisms are still elusive; among them, a wide range of excretory and secretory products from schistosomes may significantly modulate host’s immune responses [11, 12].

Helminth infection has a negative correlation with the severity of allergic airway inflammation. Several species of helminthes including *S*. *japonicum* have been shown to down-modulate airway hyperresponsiveness in human and murine models [13–15]. The interaction between helminthes infection and airway inflammation is affected by various factors, such as infection phases, parasite loads and species of helminthes [16]. Asthma is a chronic airway inflammation, in which Th2 cells are generally believed playing important roles in its pathogenesis. Allergic asthma accounts for about 80% of asthma cases, which is characterized by Th2 cytokines interleukin 4 (IL-4), IL-5 and IL-13 dependent increased immunoglobulin E (IgE) serum levels, lung eosinophilia, airway hyperresponsiveness and goblet cell metaplasia, respectively [17–19]. Recently, it is evident that other T cell subsets, such as regulatory T (Treg) [20, 21] and Th17 cells [22, 23] are actively involved in the airway hyperresponsiveness. Patients with severe asthma are often difficult to treat and resistant to the steroid therapy [22]. Th17 cells and neutrophils may play central roles in the pathogenesis of severe asthma [22]. It is generally believed that eosinophilic inflammation is mainly mediated by Th2 cells, whereas neutrophilic inflammation is a hallmark of Th17 responses [24]. Murine asthma models have been of invaluable importance in elucidating the pathogenesis of this disease [19]. In addition to classical ovalbumin-induced Th2 and eosinophils dominant allergic airway inflammation model [25–27], our laboratory recently established the house dust mite (HDM)-induced murine asthma model, in which Th17 and neutrophils are dominant responders, mimicking severe asthma [19, 28–31].

In this study, we investigated the impact of *S*. *japonicum* infection on HDM-induced allergic airway inflammation. We designed early and late HDM-immunization regimens to fit the time points before or on/after schistosome-induced immunomodulation initiated. We found that *S*. *japonicum* infection, at both early and later phases, was able to significantly inhibit the airway hyperresponsiveness. This inhibition may be related to upregulated Treg cells during *S*. *japonicum* infection.

## Materials and methods

### Ethics statement

The care and handling of the animals in this study were in strict accordance with the Guidelines for the Care and Use of Laboratory Animals at Jiangsu Institute of Parasitic Disease (JIPD). This study was prospectively approved by the Institutional Animal Care and Use Committee of JIPD (Permit Number: [2013] 016). All efforts were made to minimize animal suffering including the use of anesthesia (sodium pentobarbital, 50 mg/kg, intraperitoneally) for the administration of HDM. Mice were monitored daily by the staff of the animal facility. The human endpoint was used for mice that lost 25% or more of their initial body weight during the study. When the animals met the criteria, or at the experimental endpoint, they were euthanized under excess isoflurane anesthesia followed by cervical dislocation according to institutional guidelines.

### Mice and Parasitology

Female C57BL/6 mice (6–8 wks) were purchased from the College of Veterinary Medicine, Yangzhou University, China, and maintained under specific pathogen-free condition at JIPD. *Schistosoma japonicum* (Chinese mainland strain) were obtained from JIPD. Mice were percutaneously infected with 15 cercariae of *S*. *japonicum* (S.j.) 3 or 5 wks prior to the induction of allergic airway inflammation.

### HDM-induced allergic airway inflammation

**Fig 1:**
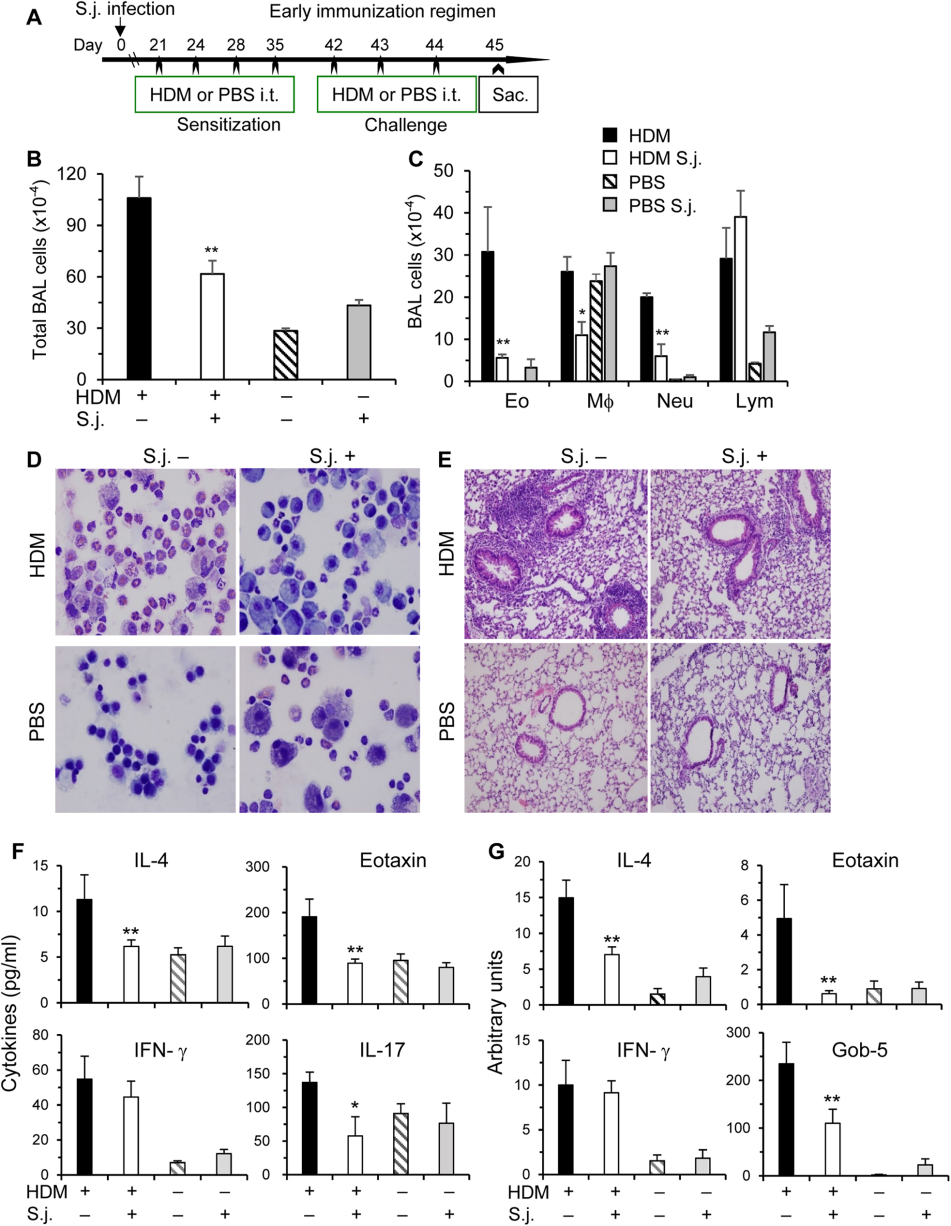
*S*. *japonicum* infection inhibits HDM-induced airway inflammation at early phase. (**A**) To induce allergic airway inflammation, both infected and uninfected C57BL/6 mice were inoculated intratracheally (i.t.) with HDM. In the early immunization regimen, HDM inoculations started at 3 wks post the infection with *S*. *japonicum* (S.j.). Control groups were inoculated i.t. with PBS alone. Mice were sacrificed 24 h after the last challenge. (**B**) The total cells in BAL fluids were counted by using a hemacytometer. (**C**) Differential cell counts of >400 cells were performed on cytospins stained with Kwik-Diff. The numbers of eosinophils (Eo), macrophages (Mϕ), neutrophils (Neu), and lymphocytes (Lym) in BAL are shown. (**D-E**) Representative Kwik-Diff staining of BAL cells and H&E staining of lung tissue sections. (**F**) Cytokine levels in BAL fluids were determined by ELISA. (**G**) Total RNA from the right lower lobe of the lungs was extracted for real-time PCR analysis for IL-4, eotaxin, IFN-γ and Gob-5. Data are normalized to an 18S reference and expressed as arbitrary units. Results are expressed as mean+SE (B-C, F-G), representative of two independent experiments (n = 5–10 per group). Compared to uninfected and HDM immunized mice, *p<0.05; **p<0.01.

**Fig 2:**
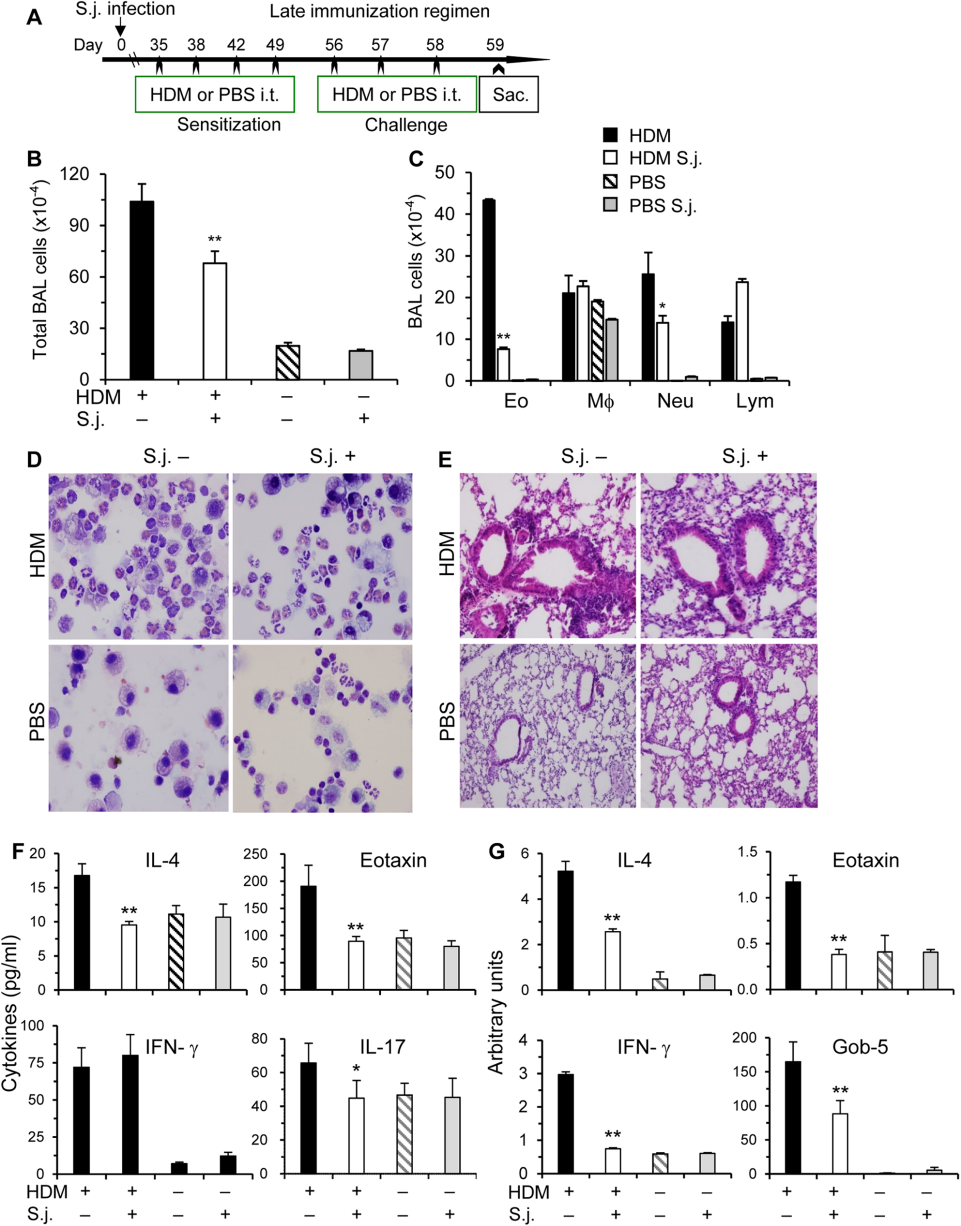
*S*. *japonicum* infection inhibits HDM-induced airway inflammation at late phase. (**A**) In the late immunization regimen, HDM inoculations started at 5 wks post infection. The experiments were performed similarly as Fig 1. Total and differential cell counts of BAL cells (**B-C**), representative Kwik-Diff staining for BAL cytospins and H&E staining of lung tissue sections (**D-E**), cytokine levels in BAL fluids (**F),** and mRNA levels in the lungs (**G**) are shown. Results are representative of two independent experiments (n = 5–9 per group). Compared to uninfected and HDM immunized mice, *p<0.05; **p<0.01.

To induce allergic airway inflammation, infected and uninfected mice were inoculated intratracheally (i.t.) with house dust mite (HDM) (*Dermatophagoides pteronyssinus*) extract (Stallergenes Greer, Lenoir, NC), starting at 3 wks (early phase) or 5 wks (late phase) post infection. The immunization regimens included 4 times of sensitizations (15 μg of HDM in 40 μl PBS, i.t. per time), and 3 times of challenges, lasting for 24 days (Fig 1A and Fig 2A). Control groups of both infected and uninfected mice were inoculated i.t. with PBS alone. Mice were sacrificed 24 h after the last challenge. Their bronchoalveolar lavage (BAL) fluids were aspirated and centrifuged. Total cells in the pellet were counted by using a hemacytometer. Differential cell counts on >400 cells were performed on cytospins stained with Shandon Kwik-Diff Stain kit (Thermo Scientific, Rockford, IL). The BAL fluid from each mouse was concentrated to 0.5 ml by centrifugation with an Amicon Ultra-4 filter unit (Millipore, Billerica, MA) for determinations of cytokines by ELISA. For lung histology, the lower lobe of the right lung was fixed with 4% paraformaldehyde overnight, dehydrated, embedded in paraffin, cut into 4 μm sections, and processed for hematoxylin/eosin (H&E) staining. Lung tissue mRNA was analyzed by Real-time PCR. Serum levels of various HDM-specific antibodies were measured by ELISA with the use of biotinylated goat anti-mouse IgE (BD Bioscience, San Jose, CA), IgM, IgG1, IgG2a, and streptavidin-HRP (Southern Biotech, Birmingham, AL).

### Cytokine assay

Cytokines in the culture supernatants and BAL fluids were measured by enzyme-linked immunosorbent assay (ELISA). IL-4, IL-10 and IFN-γ were measured with OptEIA kits (BD Bioscience); IL-13, IL-17 and eotaxin were measured with DuoSet ELISA kits (R&D Systems, Minneapolis, MN). ELISA plates were developed with TMB substrate (BD Bioscience), and read with a microplate reader. Cytokine mRNA levels were measured by real-time quantitative PCR.

### In vivo cytokine capture assay (IVCCA)

The in vivo cytokine levels for IL-4, IL-10 and IFN-γ were detected by IVCCA [32, 33], following the manufacturer’s protocols (BD Bioscience). Mice were injected with biotinylated neutralizing monoclonal antibodies to capture the corresponding cytokines, and bled 2–4 h later. IVCCA facilitates measurement of cytokines in serum by increasing their in vivo half-lives. By this way, the sensitivity of in vivo cytokine assays increases for at least 30- to 1,000-fold [32]. IVCCA for IL-17 was developed in our laboratory with the use of biotinylated anti-mouse IL-17 (BD Bioscience), recombinant murine IL-17 and IL-17 ELISA kit (R&D Systems).

### Flow cytometry

Cells were incubated with anti-CD16/32 (2.4G2) (BD Bioscience) to block FcγR II/III, and then stained with various conjugated antibodies as indicated. BD Cytofix/Cytoperm kit (BD Bioscience) was used for intracellular cytokine staining and Treg staining kit (e-Bioscience, San Diego, CA) was used for detection of Treg cells, following the manufacturer’s instructions Stained cells were analyzed by FACSVerse with FACS Suite (BD Bioscience) or FCS Express (De Novo Software, Los Angeles, CA) software.

### Real-time PCR

Total RNA was extracted from lung tissues with the RNeasy Mini Kit (Qiagen, Valencia, CA), and cDNA was prepared by using the High-Capacity cDNA Reverse Transcription Kit (Applied Biosystems, Foster City, CA). Quantitative PCR was performed with the Platinum SYBR Green qPCR SuperMix-UDG w/RO or TaqMan Gene Expression Master Mix (Life Technologies, Carlsbad, CA) on a Light Cycler 480 II (Roche, Indianapolis, IN). The data were normalized to the 18S reference. Primers for IL-4, IL-5, IL-13, eotaxin, MUC-5AC, and Gob-5 were designed with OLIG 4.0 software as reported [26].

### Statistical analysis

All experimental data were analyzed and compared for statistically significant differences using two-tailed Student’s *t* or Mann-Whitney *U* test, and a *P* value of < 0.05 was considered significant.

## Results

### *S*. *japonicum* infection inhibits HDM-induced airway inflammation

Helminth infection may significantly modulate host’s immune responses. We evaluated the impact of *S*. *japonicum* infection on the allergic airway inflammation induced by HDM. Mice were repeatedly inoculated HDM intracheally for about 4 wks, starting at 3 or 5 wks post infection respectively. The early (starting at 3 wks) or late (5 wks) HDM-immunization regimens were designed trying to fit the time points before or on/after schistosome-induced immunomodulation initiated. Airway inflammation was evaluated by quantitation of total cells in the bronchoalveolar lavage (BAL) fluids, the differential cell counts of BAL cells by Kwik-Diff staining, and infiltrating cells in lung tissues by H&E staining.

In the early immunization regimen (Fig 1A), total numbers of BAL cells in uninfected mice inoculated with HDM had over 3~4-fold increases as compared to PBS-inoculated control mice (Fig 1B). By differential counts with Kwik-Diff Staining, a mixed cell infiltration was observed, mainly including eosinophils, macrophages, neutrophils and lymphocytes (Fig 1C and 1D). However, *S*. *japonicum* infection led to a dramatic reduction in total numbers of BAL cells, particularly eosinophils and neutrophils, whereas, the proportions of macrophages were relatively increased. In the lungs, uninfected mice showed massive perivascular and peribronchial infiltration of inflammatory cells upon HDM-challenge as revealed by lung histological analysis. However, this inflammatory response was markedly attenuated in mice after *S*. *japonicum* infection (Fig 1E). We reasoned that this infection-induced inhibitory effect on allergic airway inflammation might be attributable to altered immune responses of various T helper (Th) subsets. In the following experiments, we determined effector cytokines of Th1, Th2 and Th17 cells. IL-4, a representative Th2 cytokine, was significantly reduced from infected mice in BAL fluids and lungs at protein (Fig 1F) and mRNA levels (Fig 1G), respectively. Similarly as IL-4, Th17 effector cytokine IL-17 was downregulated in infected mice in BAL fluids (Fig 1F). On the other hand, Th1 cytokine IFN-γ was comparable in both BAL fluids and lungs between the infected and uninfected mice (Fig 1F and 1G).

Hypersecretion of mucus plays an important role in the pathogenesis and severity of asthma [25]. Gob-5 gene has been suggested to promote the synthesis of mucin glycoproteins by upregulation of MUC-5AC gene expression [34]. Gob-5 was dramatically increased in HDM-inoculated uninfected mice (Fig 1G), whereas, over half of this increase was lost in the infected mice. Eotaxin, by acting on CCR3, selectively recruit eosinophils from the airway microvessels into the lung tissue, which plays a central role in the onset of allergic asthma [35]. This important chemokine was significantly reduced in the BAL fluid in *S*. *japonicum* infected mice (Fig 1F).

The findings from the late immunization regimen (Fig 2) basically followed the similar patterns as the early immunization regimen. Airway inflammation, Th2 and Th17 effector cytokines were consistently downregulated in infected mice in the early as well as late HDM-immunization regimens. These data suggest that *S*. *japonicum* infection significantly affects host’s allergic airway inflammation.

### HDM-specific IgE downregulated upon *S*. *japonicum* infection

**Fig 3:**
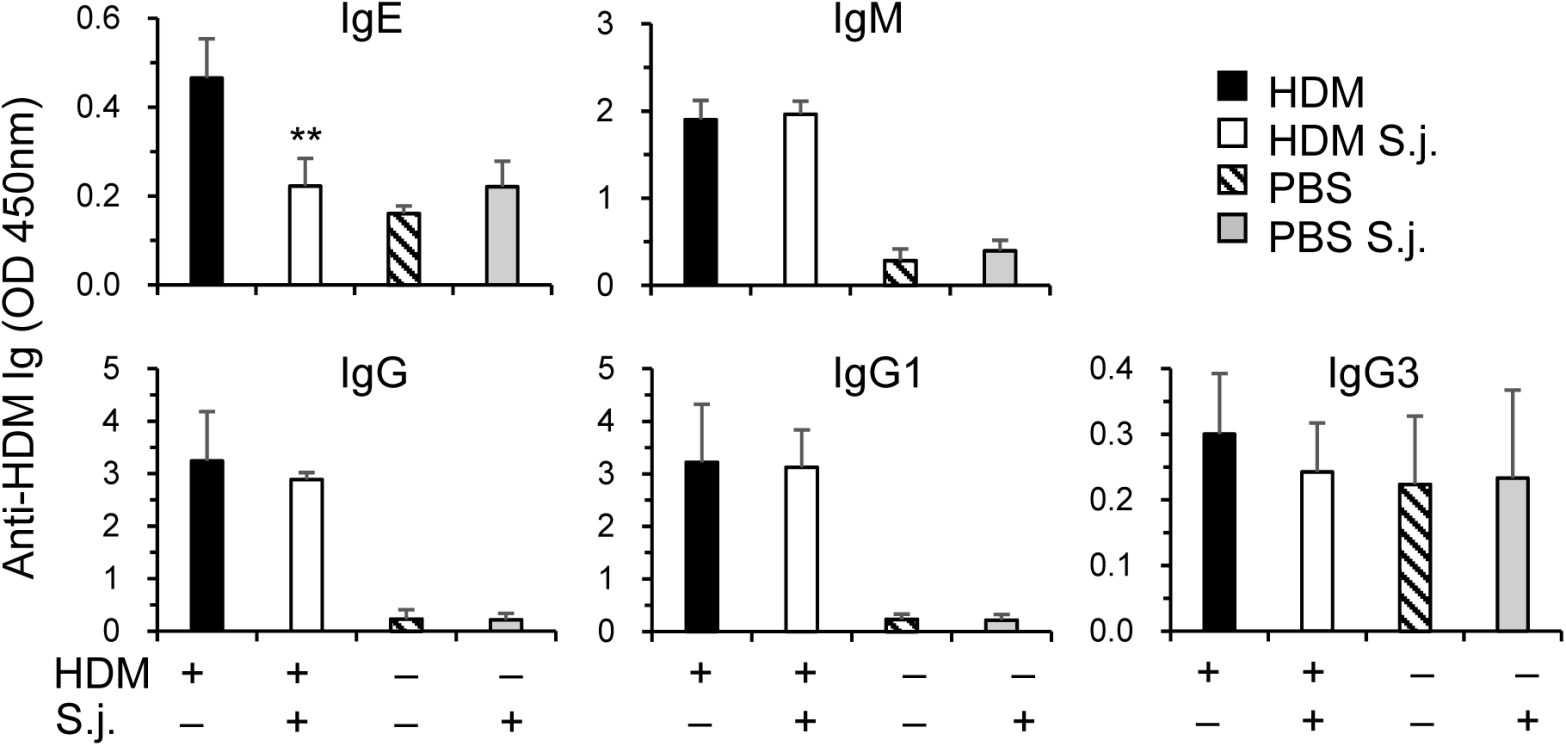
Serum OVA-specific IgE is reduced in *S*. *japonicum* infected mice. In the same experiments as Fig 2, serum levels of OVA-specific IgE, IgM, IgG and IgG subclasses were assayed by ELISA (mean+SE). **p<0.01 compared to uninfected and HDM immunized mice.

Apart from cellular responses in airway, humoral systemic responses to HDM are also important for asthma pathogenesis and development [25, 36]. IgE is an important mediator of allergic airway inflammation, which is related to Th2 cytokine IL-4 [36]. We found that serum levels of HDM-specific IgE were significantly reduced in *S*. *japonicum* infected mice. However, HDM-specific IgM, total IgG, and its subclasses IgG1, IgG3 did not differ significantly between infected and uninfected groups (Fig 3).

### Dynamics of in vivo cytokine levels during *S*. *japonicum* infection

**Fig 4:**
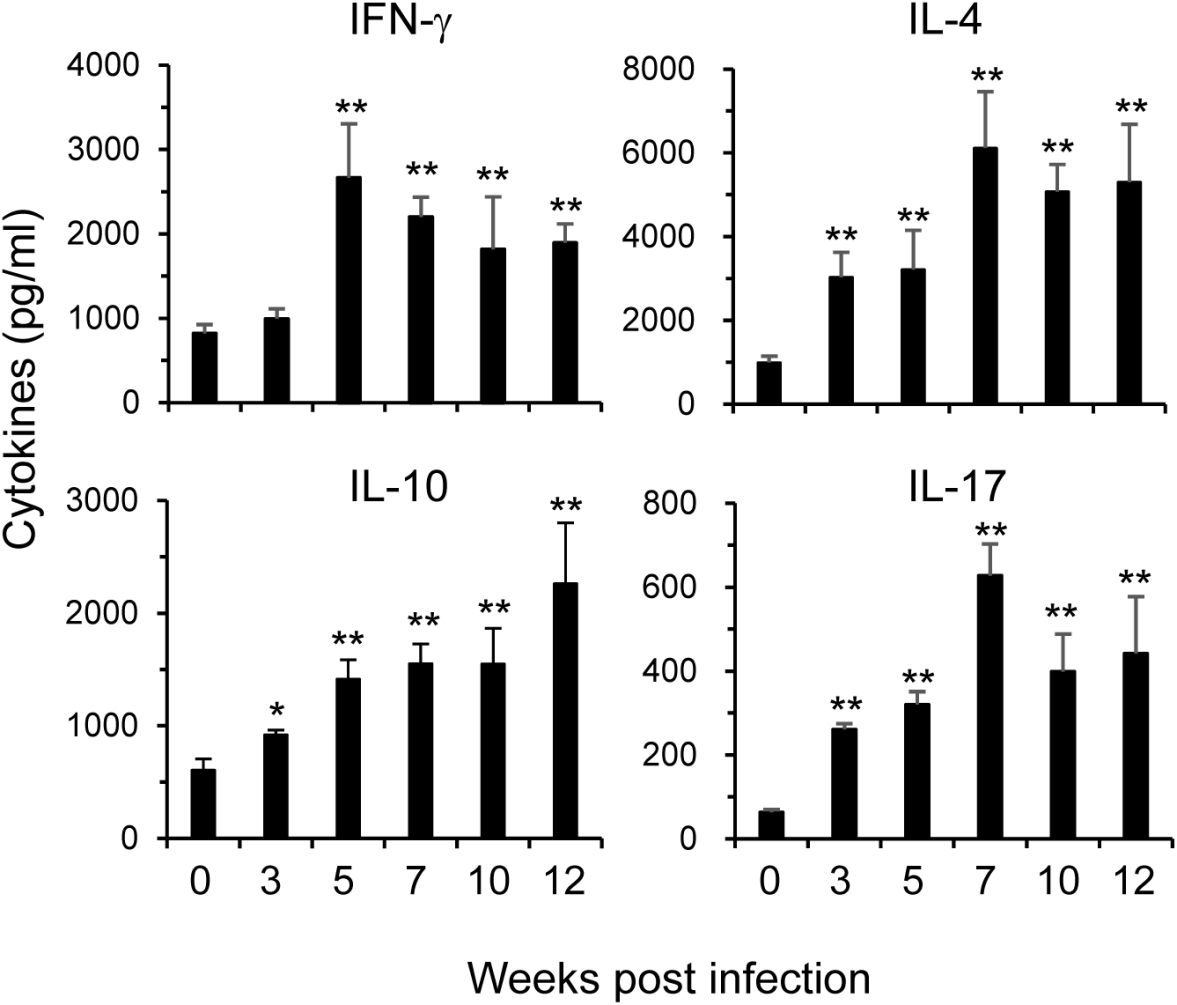
Cytokine dynamics in vivo post infection. C57BL/6 mice were infected with *S*. *japonicum*. The levels of IFN-γ, IL-4, IL-10, and IL-17 cytokines in the peripheral blood were assayed at indicated times by IVCCA. Results are representative of two independent experiments (n = 5–9 per group). Compared to uninfected mice, **p<0.01.

To begin to understand the underlying mechanisms of *S*. *japonicum* infection-induced inhibition to airway inflammation, we next studied the in vivo dynamics of various Th effector cytokines during the infection. Because most cytokines are utilized, catabolized, or excreted shortly after they are produced in vivo, it has been difficult to directly measure in vivo cytokine production [32, 33]. IVCCA was developed to determine the circulating levels of various cytokines. Once *S*. *japonicum* infected the host, a wide range of excretory and secretory products from schistosomes may downregulate host’s immune responses, including differentiation and function of various Th subsets. We monitored the in vivo dynamics of effector cytokines for Th1, Th2, Treg and Th17 upon infection progression. Serum levels of IFN-γ, IL-4, IL-10 and IL-17 were determined by IVCCA at 0, 3, 5, 7, 10 and 12 wks post infection (Fig 4).

Serum level of IFN-γ started to increase at 3 wks, peaked at 5 wks post infection; then, significantly downregulated but remained at high levels compared to prior infection (0 wk). IL-4 reached its peak at 7 wks post infection; then gradually decreased but kept at high level till 12 wks after infection. On the other hand, serum level of IL-10 kept rising and maintained a plateau from 5 to 12 wks post infection. IL-17 in sera reached its peak at 7 wks after infection then gradually reduced but remained at elevated level 12 wks post infection. These data, for the first time, revealed the in vivo dynamics of cytokine profiles during *S*. *japonicum* infection.

**Fig 5:**
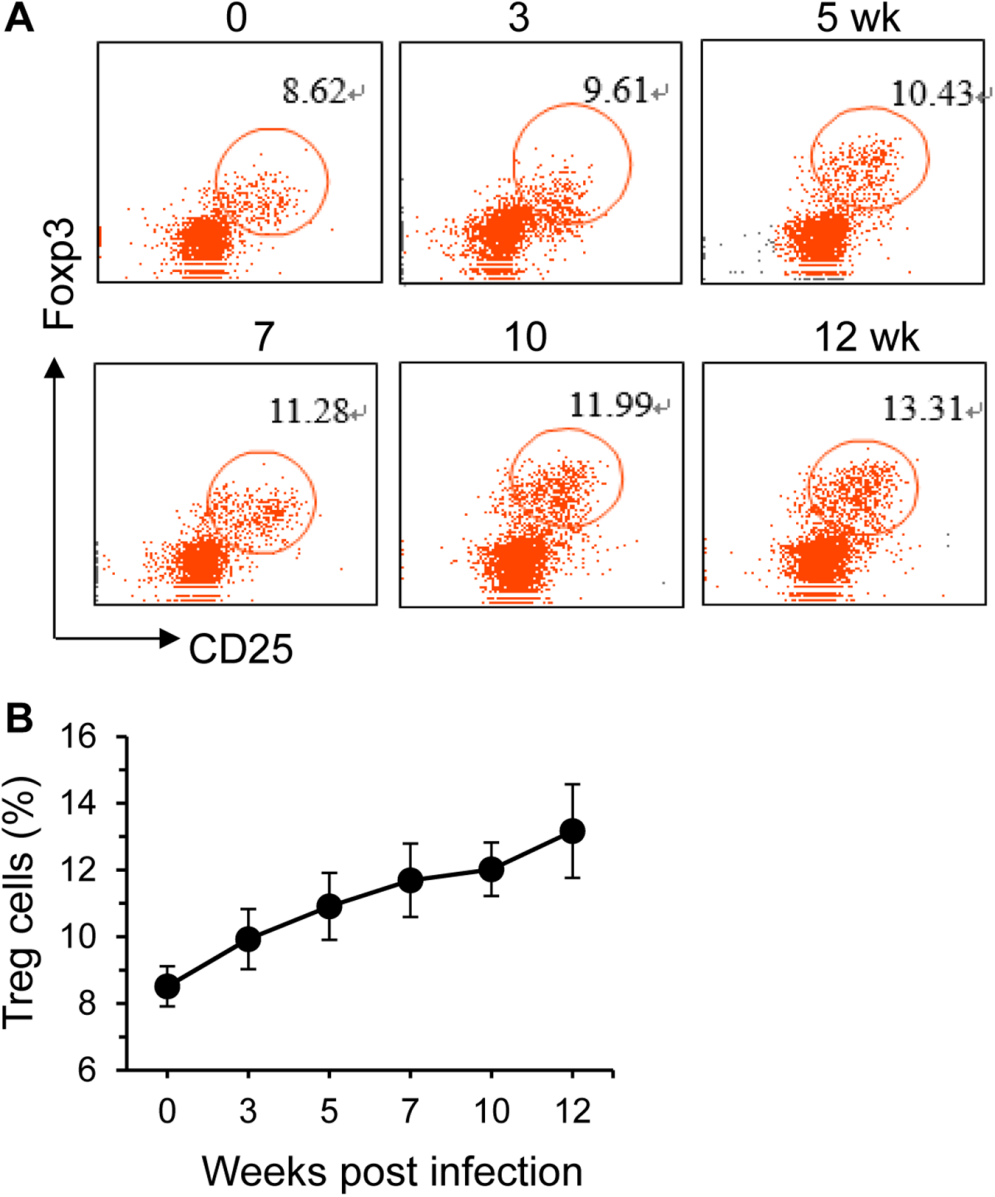
Treg cells expand after *S*. *japonicum* infection. Treg cells in the spleens were determined by Flow cytometry gated on CD4^+^ cells. Percentages of Treg cells (CD4^+^CD25^+^Foxp3^+^) cells are shown in representative dot plots (**A**), and summarized in a graph (**B**). Results are representative of two independent experiments (n = 5–6 per group).

We also determined the dynamic changes of Treg cells in the spleens by FACS. These cells expanded upon *S*. *japonicum* infection (Fig 5). The main Treg effector cytokines are TGF-β1 and IL-10 [37]. The dynamics of Treg cells in the spleens were consistent with the in vivo IL-10 changes by IVCCA (Fig 4).

## Discussion

Immunomodulation by parasitic infection has become a hot research topic recently. The ability of infectious agents to regulate their host’s immune system is becoming an interesting research attraction [6]. Ample evidences have now support the notion that both microbes and parasites can effectively modulate host’s immune responses to alleviate allergic and autoimmune disorders [3, 6]. Some helminthes seem to have anti-allergic or anti-inflammatory effects in humans. Experimental evidences have also shown the significant suppression for the development of airway hyperresponsiveness in mice infected with numerous helminths, including *S*. *japonicum* [15]. Infection by this parasite causes severe immunopathogenic injuries to the host; on the other hand, it may open a new avenue and idea to develop novel therapies to autoimmunity and allergy based on parasitic products and immunomodulation mechanisms according to the “hygiene hypothesis”.

Asthma is traditionally believed as Th2 cells and eosinophils-dominant chronic airway inflammation. Th2 cytokines IL-4 and IL-13 can activate epithelial and endothelial cells and fibroblasts to produce eotaxin, a chemotaxin important for recruiting eosinophil from the airway microvessels into the lung tissue [35]. Recently, compelling evidences have demonstrated that Th17 cells are a major player to the pathogenesis of allergic airway inflammation, especially to severe asthma [22, 23, 29, 38–40]. The latter is often induced by infection and resistant to steroid therapy in patients [41].

By intracheal inoculations of HDM, we developed a murine asthma model, in which Th17 cells and nertrophil are dominant responders, mimicking severe asthma [19, 22]. With this model, we investigated the impacts of *S*. *japonicum* infection on the allergic airway inflammation. Our previous study revealed that IL-4 reached a peak at 7 wks post infection, then declined steadily reaching the base line by week 20 post-infection from spleen cells of *S*. *japonicum* infected mice when stimulated with the soluble egg antigens [9]. The in vivo IL-4 level also peaked at 7 wks post infection, while, IFN-γ began to decline at this time point (Fig 4). It was found that egg deposition was the major factor driving Th2 responses, depressing Th1 cytokine expression as well as T-cell proliferation in *S*. *japonicum*-infected mice [9]. Therefore, we designed the early (starting at 3 wks) and late (5 wks) HDM-immunization regimens trying to fit the time points before or on/after schistosome-induced immunomodulation initiated in the current study. It was found that *S*. *japonicum* infection, at both early and later phases, was able to significantly inhibit the airway hyperresponsiveness. Th2 and Th17 effector cytokines in the BAL fluids and lungs were markedly downregulated. *S*. *japonicum* infection may inhibit eosinophil infiltration via the downregulation of eotaxin and IL-4 in the lung and BAL; whereas, IL-17 downregulation may contribute to the ameliorated neutrophil infiltration in airways. Not only cytokine secretions were inhibited, serum HDM-specific IgE was also inhibited by the infection, which may contribute to the reduced allergic airway inflammation too, as IgE plays an important role in the immunopathogenesis of asthma by activating mast cells and basophils through its binding to Fcε Receptors, etc [35]. On the other hand, HDM-specific IgG and IgM did not alter by the infection. Therefore, the reduced serum level of HDM-specific IgE was not caused by the generalized inhibition of B cells. Instead, it may be related to the impaired Th2 function, as IL-4 preferentially induces Ig isotype switching to IgE [26, 36].

The underlying mechanisms by which *S*. *japonicum* infection alleviates allergic airway inflammation are yet to be elucidated. We found that Treg cells in the spleens were continuously upregulated upon infection. TGF-β1 and IL-10 are the main Treg effector cytokines. In vivo IL-10 level was raised 5 wk post infection by IVCCA assay. These data are consistent with other reports that Treg cells participate in the regulation of schistosomiasis not only locally, but also systemically [42–44]. We found a systemic increase of IL-17 in the serum post *S*. *japonicum* infection (Fig 4); whereas, airway IL-17 in the BAL fluids was reduced (Fig 1F and Fig 2F). The mechanisms for this discrepancy remain elusive. The upregulation of systemic IL-17 might reflect the direct responses of Th17 cells to the parasite antigen stimulation; whereas, the inhibited BAL IL-17 secretion to HDM might be caused by activation of Treg cells in the airways. Further studies are needed to identify the components from schistosomes including their excretory and secretory products, which possess the ability to directly modify the responses of different Th subsets, such as Treg, Th17 cells.

In summary, our findings indicated that *S*. *japonicum* infection was able to effectively inhibit house dust mites-induced allergic airway inflammation. This inhibition may be related to the upregulated Treg cells upon infection. More in depth investigations are need to elucidate the underlying cellular and molecular mechanisms.
