# Correction: *Schistosoma japonicum* infection downregulates house dust mite-induced allergic airway inflammation in mice

**DOI:** 10.1371/journal.pone.0190996

**Published:** 2018-01-05

**Authors:** Sugan Qiu, Xiaolin Fan, Yingying Yang, Panpan Dong, Wei Zhou, Yongliang Xu, Yonghua Zhou, Fukun Guo, Yi Zheng, Jun-Qi Yang

The authors would like to correct Fig 2E, as an error was introduced in the preparation of this figure for publication. In Fig 2E, the incorrect image was used for “S.j.-” in the “PBS” row. The image incorrectly appears as a duplicate of the “S.j.+” image in the PBS row of Fig 1E. The authors have provided a complete, corrected version of Fig 2 here.

**Fig 2:**
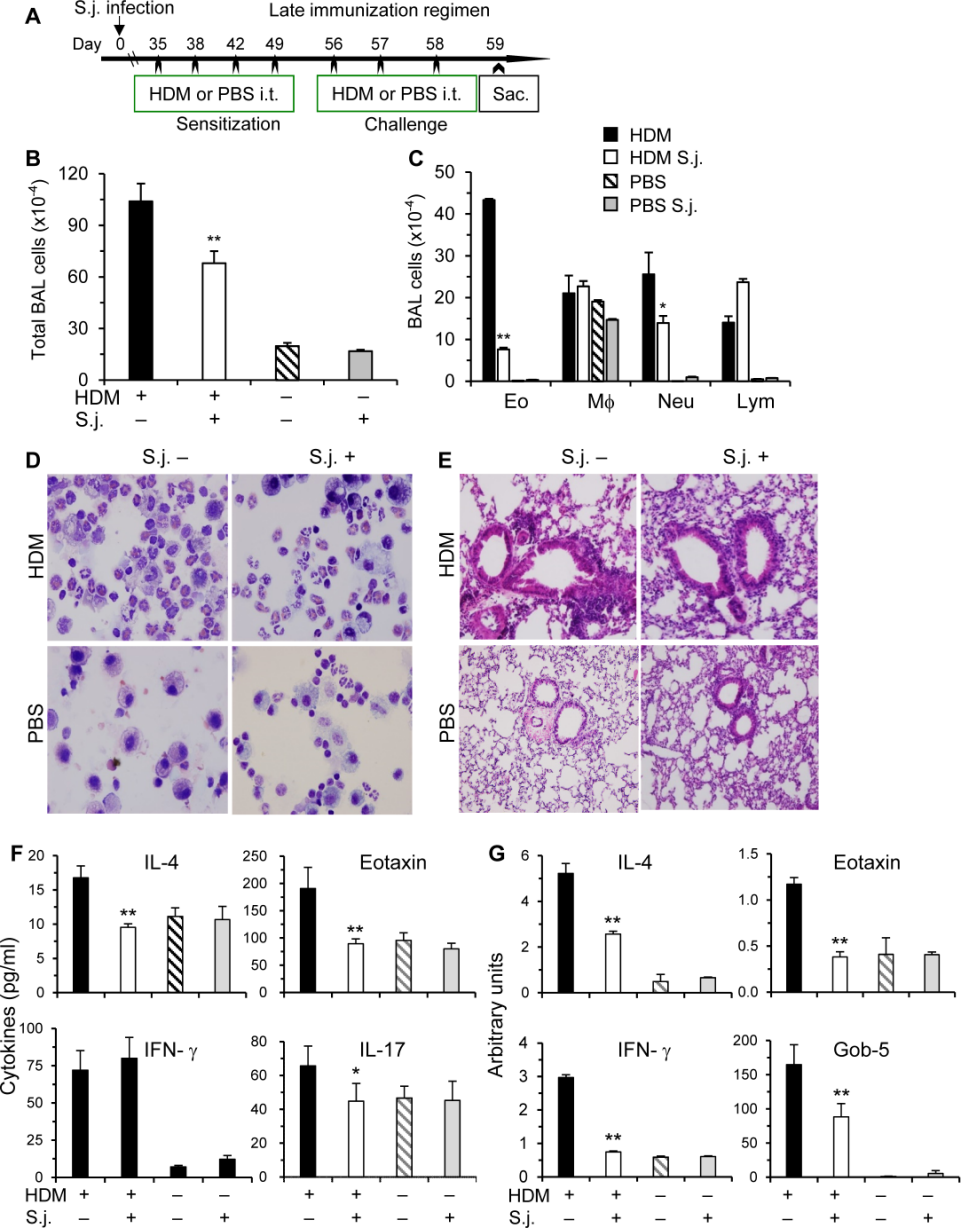
*S*. *japonicum* infection inhibits HDM-induced airway inflammation at late phase. **(A)** In the late immunization regimen, HDM inoculations started at 5 wks post infection. The experiments were performed similarly as Fig 1. Total and differential cell counts of BAL cells **(B-C)**, representative Kwik-Diff staining for BAL cytospins and H&E staining of lung tissue sections **(D-E)**, cytokine levels in BAL fluids **(F)**, and mRNA levels in the lungs **(G)** are shown. Results are representative of two independent experiments (n = 5–9 per group). Compared to uninfected and HDM immunized mice, *p<0.05; **p<0.01.

The authors confirm that these changes do not alter their findings.

There is an error in the caption for Fig 3. Please see the complete, correct Fig 3 caption here.

**Fig 3:**
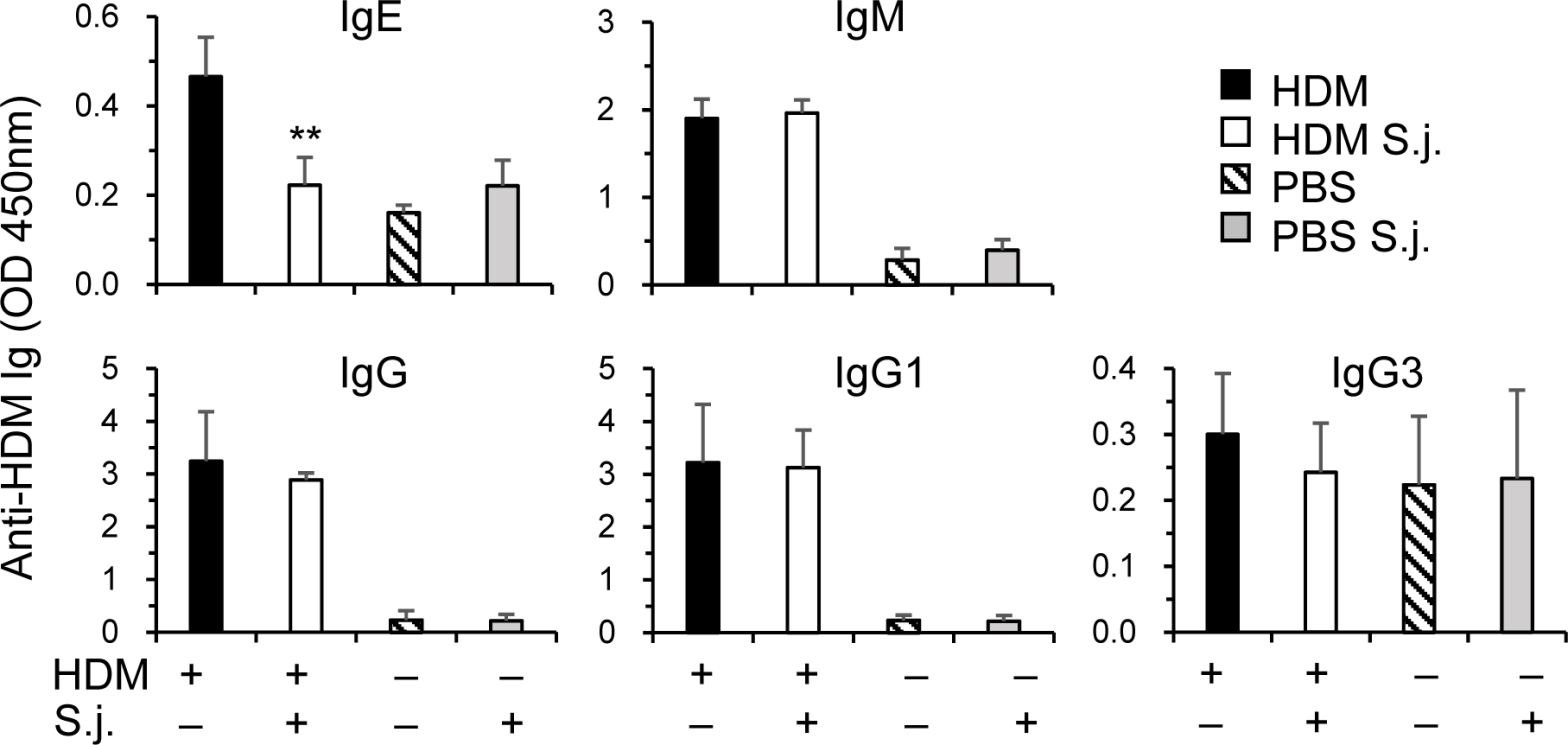
Serum HDM-specific IgE is reduced in *S*. *japonicum* infected mice. In the same experiments as Fig 2, serum levels of HDM-specific IgE, IgM, IgG and IgG subclasses were assayed by ELISA (mean+SE). **p<0.01 compared to uninfected and HDM immunized mice.
